# Transcriptome changes in apple peel tissues during CO_2_ injury symptom development under controlled atmosphere storage regimens

**DOI:** 10.1038/hortres.2015.61

**Published:** 2015-12-23

**Authors:** Franklin T Johnson, Yanmin Zhu

**Affiliations:** 1 Department of Horticulture, Washington State University, Pullman WA 98664, USA; 2 Tree Fruit Research Laboratory, USDA-ARS, Wenatchee, WA 98801, USA

## Abstract

Apple (*Malus* × *domestica* Borkh.) is one of the most widely cultivated tree crops, and fruit storability is vital to the profitability of the apple fruit industry. Fruit of many apple cultivars can be stored for an extended period due to the introduction of advanced storage technologies, such as controlled atmosphere (CA) and 1-methylcyclopropane (1-MCP). However, CA storage can cause external CO_2_ injury for some apple cultivars. The molecular changes associated with the development of CO_2_ injury are not well elucidated. In this study, the global transcriptional regulations were investigated under different storage conditions and during development of CO_2_ injury symptoms on ‘Golden Delicious’ fruit. Fruit peel tissues under three different storage regimens, regular cold atmosphere, CA and CA storage and 1-MCP application were sampled at four storage durations over a 12-week period. Fruit physiological changes were affected differently under these storage regimens, and CO_2_ injury symptoms were detectable 2 weeks after CA storage. Identification of the differentially expressed genes and a gene ontology enrichment analysis revealed the specific transcriptome changes associated with each storage regimen. Overall, a profound transcriptome change was associated with CA storage regimen as indicated by the large number of differentially expressed genes. The lighter symptom was accompanied by reduced transcriptome changes under the CA storage and 1-MCP application regimen. Furthermore, the higher enrichment levels in the functional categories of oxidative stress response, glycolysis and protein post-translational modification were only associated with CA storage regime; therefore, these processes potentially contribute to the development of external CO_2_ injury or its symptom in apple.

## Introduction

Apple (*Malus* × *domestica* Borkh.) is one of the most widely cultivated tree fruit.^1^ Postharvest storability of apple fruit is a critical component to the success of the apple fruit industry. Fruit of many apple cultivars can be stored for an extended period with minimal loss of quality due to the advancements in storage technology. These technologies include controlled atmosphere (CA) storage regimen and the application of ethylene action blocker 1-methylcyclopropane (1-MCP).^2–4^ With regard to the role of ethylene-regulated apple fruit ripening process regulating apple fruit ripening process, proper strategies to suppress or delay ethylene production, and therefore fruit ripening progression, are the core of postharvest quality management strategies of apple fruit storage.

CA storage, by storing fruit and vegetable in an atmosphere with elevated CO_2_ and reduced O_2_ levels, suppresses ethylene production, slows down the fruit ripening process and enhances fruit storability.^5–8^ However, CA storage regimen is known to cause physiological disorders such as internal or external CO_2_ injury to the fruit of some apple cultivars.^9–11^ The typical symptoms of external CO_2_ injury are irregular-shaped brown-to-bronze–colored lesions with defined edges on the fruit peel tissue, and the injury can extend from the peel into the outer cortex tissues.^11, 12^ Decreasing the levels of CO_2_ during the early period of CA storage or delaying the application of CA storage after harvest have been shown to be able to reduce injury risk.^10, 11, 13, 14^ The SmartFresh^®^ formulation of 1-MCP, functioning as an ethylene response inhibitor by irreversibly binding to the ethylene receptor,^15^ has been widely used in postharvest anagement practices to improve the storability of fresh fruit.^2, 16^ Nevertheless, application of 1-MCP has been indicated in increasing the development of CO_2_ injury to the fruit of some cultivars.^17–19^ The incidence of CO_2_ injury can significantly vary year to year even for the same cultivar, indicating the possible preharvest factors on disorder risk. Currently, the understanding of the underlying molecular regulations associated with CO_2_ injury development is limited.^20^

To understand the underlying molecular mechanisms potentially associated with the development of apple CO_2_ injury symptoms, transcriptome profiling is one of the available approaches for this less investigated area. Transcriptional regulation is the first step in the conversion of genome-encoded information into the physiological changes.^21^ Transcriptome analysis aims to simultaneously monitor the regulations of entire inventory of transcripts in a cell or tissue under specific conditions.^22, 23^ High-throughput and high-resolution RNA sequencing (RNA-seq) technology has become a mainstay for plant transcriptomic analyses within the past decade.^24–26^ The objective of this study was to identify the transcriptome changes in the peel tissue of ‘Golden Delicious’ apple fruit under three different storage regimens during a 12-week storage period. Specifically, our goals are to identify genes and pathways associated with an individual storage regimen, i.e. RA (regular atmosphere), CA (controlled atmosphere) and CAP (CA plus 1-MCP treatment). The results from this study will be the foundation for future studies aiming to identify specific genes and biochemical pathways that are responsible for the development of apple external CO_2_ injury.

## Materials and methods

### Fruit sample and postharvest storage regimens

‘Golden Delicious’ apples with uniform size and appearance were harvested from a commercial orchard near Quincy, WA, during the 2011 apple-growing season. The trees were 35 years old and had been grafted on M111 rootstocks. Fruits were stored following three storage regimens, i.e. RA: regular atmosphere; CA: 5% CO_2_ and 1% O_2_ CA; or CAP: 1-MCP treatment before CA storage. All fruits except those for CAP storage regimen were placed at room temperature overnight, until the completion of 1-MCP treatment. Then all fruits were put into 0.5 °C storage chambers at the same time. At each sampling date, i.e. 0 (before storage), 2, 4, 8 and 12 weeks of storage, 144 apples for each storage type were evaluated for CO_2_ injury. Fruit physiological data per storage regimen and duration were collected using a set of 15 apples. Peel tissues were collected from a set of 15 apples and pooled into three biological replicates of five apples. The tissues with symptoms were also included with the portion representing percentages of the whole sample at the specific duration. The peel tissues were immediately flash-frozen in liquid nitrogen (Norco Inc., Wenatchee, WA) and then stored at −80 °C before RNA isolation or metabolomic analysis.

### Fruit physiological changes during storage

Fruit firmness was measured for each storage type at each time point using a Mohr Digi-Test® MDT-2 instrument (Mohr and Associates, Richland, WA). On a pared surface of each apple, using an 11.1-mm-diameter probe, a computerized texture analyzer calculated the MDT-2 results. The A1 measurement of fruit firmness was converted from Newton into pound (lb). Internal ethylene concentration (IEC) was measured for each storage type at each time point by withdrawing a sample of air from the air space within the apple core. The air space was reached by puncturing the calyx end with a 3.81-cm probe with rubber stopper placed on the large aperture side of the probe. The rubber stopper attached to the probe was pierced with a 25-G 5/8 (0.5 mm × 16 mm) needle connected to a 1-ml latex-free syringe. A Hewlett Packard 5880A gas chromatograph equipped with a Porapak Q column (0.3-cm internal diameter × 30 cm, 80–100 mesh) and a flame ionization detector was used to measure IEC. Injector, oven, and FID temperatures were 50°, 50° and 100 °C. The flow rates for N_2_ carrier gas, H_2_, and air were 5, 30, and 300 ml min^−1^, respectively. The Hewlett Packard 5880A gas chromatograph was calibrated to a 9.606 p.p.m. (±0.27 p.p.m. accuracy of analysis) sample of ethylene calibration gas (Scott Speciality Gases, Applied Science Labs, Deerfield, IL). A 1-ml volume of air was taken from the air space of the apple core, 0.5-ml volume was flushed out of the syringe and the remaining volume was used to measure IEC. Starch pattern indexing (SPI) of apple fruit was assessed for each storage type at each time point during the storage durations. SPI was visualized by staining equatorial cut sections of apples with iodine solution (40 g of potassium iodide and 10 g of iodine crystals per gallon of water). A ‘Golden Delicious’ 1–6 SPI reference chart was used to rate apples (1 = least degraded starch, 6 = most degraded starch).^27^

### RNA isolation from apple fruit peel tissue

Total RNA was isolated from the fruit peel tissue from each storage type at each time point, which was used for RNA-seq, following the method described by Gasic *et al*.^28^ Approximately 4 g of frozen peel tissue was ground to a fine powder using an analytical mill (IKA Works, Inc., Wilmington, NC), which was chilled with liquid nitrogen (Norco Inc.) prior to and during the milling process. The mill was washed with distilled water and dried between each sample. The fine-powdered peel tissue was then transferred to a 50-ml polypropylene tube containing 15 ml of RNA extraction buffer (2% CTAB-Cetyltrimethyl ammonium bromide, 2% polyvinylpyrrolidone K-30 (soluble), 100 mM Tris–HCl (pH 8.0), 25 mM EDTA-Ethylenediaminetetraacetic acid, 2 M NaCl, 0.5 g L^−1^ spermidine (free acid) and 2% (300 µL) β-mercaptoethanol) (Sigma-Aldrich, St Louis, MO). Polypropylene tubes were vortexed for 30 s and heated to 60 °C for 15 min using a hot water bath. Tubes were removed and vortexed for 30 s and then returned to the hot water bath for an additional 15 min.

After equilibrating the RNA extraction mixture, 10 ml of chloroform:isoamyl alcohol (24:1) was added to each tube, which was vortexed for 2 min and centrifuged at 10 000*g* for 10 min at 4 °C. Supernatant was transferred to clean 50-ml tubes, and to the second extract, 10-ml chloroform:isoamyl alcohol was added and centrifuged again at 10 000*g* for 10 min at 4 °C. The total collected supernatant was then transferred to 15-ml polypropylene tubes and approximately 1/3 volume of 7.5 M LiCl was added to each tube. The solution was gently mixed by inversion and then stored at 4 °C overnight. The tubes were then centrifuged at 14 000*g* for 30 min at 4 °C. The supernatant was discarded, and the pellet washed with 750 µL of 70% ethanol, which was centrifuged at 14 000*g* for 10 min at 4 °C prior to pouring off the ethanol. The tubes were placed in the hood inverted on Kimwipes for 3–5 min to dry the pellet. The total RNA pellet was dissolved in 500 µL of DEPC-Diethylpyrocarbonate water and was precipitated in 1/10 volume of 3 M sodium acetate (pH 5.5) and 2 volumes of 100% ethyl alcohol for 1–3 h at −80 °C. Tubes were centrifuged at 14 000*g* for 30 min at 4 °C, the supernatant was discarded, the pellet was washed with 70% ethyl alcohol, and the tubes were centrifuged again at 14 000*g* for 10 min at 4 °C. The supernatant was discarded, and tubes were placed in the hood inverted on Kimwipes for 3–5 min and air dried for 15–20 min. Total RNA pellets were dissolved in 30 µL of molecular biology–grade water (Sigma-Aldrich). Total RNAs from three biological replicates were pooled to represent the sample per treatment and storage duration. The total RNA was quantified using an ND-1000 Nanodrop spectrophotometer (NanoDrop Technologies, Wilmington, DE), and the RNA quality was verified by agarose gel electrophoresis.

### RNA-seq by Illumina

The quantity and quality of isolated peel RNA was examined using a Nanodrop ND-1000 (Thermo Scientific, Waltham, MA) and 2100 bioanalyzer (Agilent Technologies, Inc., Santa Clara, CA). Poly-A–containing mRNA was purified and was converted to complimentary DNA (cDNA) (TruSeq RNA sample preparation kit; Illumina, Inc., San Diego, CA). Prior to cDNA synthesis, 2 µg of total RNA was enriched for mRNA using oligo-dT–attached magnetic beads. SuperScript II reverse transcriptase (Life Technologies, Grand Island, NY) and first-strand master mix from the TruSeq kit (Illumina, Inc.) were used for first-strand synthesis under the following conditions: 25 °C for 10 min, 42 °C for 50 min, 72 °C for 15 min and then held at 4 °C. Second-strand master mix from the TruSeq kit (Illumina, Inc.) was used for second-strand biosynthesis. RNA was fragmented by using divalent cations under elevated temperature, and short fragments were removed using the AMPure XP beads. PCR enrichment was performed under the following conditions: 98 °C for 30 s; 15 cycles of 98 °C for 10 s, 60 °C for 30 s, 72 °C for 30 s; 72 °C for 5 min; and then held at 10 °C. The quality of libraries was checked with 2100 bioanalyzer and HS-DNA chip (Agilent Technologies, Inc.). The multiplex libraries (five multiplex libraries per lane) were prepared for HiSeq 2000 (Illumina, Inc.) for single-end sequencing of 101 bases without replication at the Center for Genome Research and Biocomputing in Oregon State University. Approximately 30 million raw reads per sample were produced.

### RNA-seq read alignment, normalization, annotation and contrasts

Raw reads generated by the Illumina HiSeq 2000 instrument were processed as follows. The apple (*M. domestica*) reference genome sequences^34^ consisting of 122 146 contigs were downloaded from the Genome Databases for Rosaseae (http://www.rosaceae.org). Data were analyzed using CLC Genomics Workbench v 5.5.1 (CLC GW; CLCBio, Boston, MA). The high-quality reads passing score limit (= 0.01 in CLC GW) were the input data for large gap read mapping against reference genome Md-v1.0 using the CLC Large Gap Read Mapping plugin. CLC GW reference Mapper was run with high stringency settings. The minimum length fraction was 0.75 and the minimum similarity was 0.90. These two high-stringent parameters were used in large gap read mapping of sequences against *M. domestica* v1. The limit for read unspecific match to *M. domestica* v1 was set to 5.

For transcript discovery, the CLC Transcript Discovery plugin was applied using default parameters. Kal’s test were run to identify differential expression between samples at different storage durations within a storage regimen, with the criteria of FDR <0.001 and with the detected fold change at or greater than 1.5.

### Functional annotation of the assembled transcripts

Assigning InterPro domains and retrieving associated gene ontology (GO) terms describing biological processes (BPs), molecular functions (MFs) and cellular components (CC)^29^ for genes were determined using InterProScan^30^ and in-house scripts. The genes involved in biologically relevant pathways were determined from the Kyoto Encyclopedia of Genes and Genomes (KEGG) pathway database through the KEGG Automated Annotation Server.^31^ To detect significant patterns among genes of experiments, based on their annotation, we performed a hypergeometric test on the main GO categories for BP, MF and CC, and pathway.

#### Primer design

Forward and reverse primers for the genes used in this study were designed using web-based Primer3plus software (www.bioinformatics.nl/cgi-bin/primer3plus/primer3plus.cgi) and an IDT oligo analyzer (www.idtdna.com/analyzer/Applications/OligoAnalyzer/). Where possible, an optimum annealing temperature of 60 °C, a GC content of 40–60%, an amplicon length of 150–180 bp and a primer length of 20 bp were applied. Multi-sequence alignment software ClustalW (www.ebi.ac.uk/clustalw) and BLAST (www.ncbi.nlm.nih.gov/BLAST/) were used to choose divergent regions among gene family members at or close to 3′ UTR (untranslated region) for gene-specific primer design.

#### RNA isolation and real-time quantitative reverse transcription–PCR

The frozen tissue samples were ground to a fine powder in liquid nitrogen, and total RNA was isolated following the modified method as described above. The total RNA was quantified using an ND 1000 Nanodrop spectrophotometer (NanoDrop Technologies) and the RNA quality was verified by agarose gel electrophoresis. Total RNA was treated with DNase I (Qiagen, Valencia, CA) and then purified with RNeasy cleanup columns. Two micrograms of DNase-treated RNA was used to synthesize first-strand cDNA using SuperScript^TM^ II reverse transcriptase (Invitrogen, Grand Island, NY) and poly dT (Operon, Huntsville, AL) was used as the primer. The cDNA was diluted 20 times and 0.6 µL of aliquot was used in a 15-µL quantitative PCR reaction mix: 0.45 µL SYBR Green I dye (Invitrogen), 1X iTaq buffer (Biorad, Hercules, CA), 0.2 mM dNTP (Applied Biosystem, Waltham, MA), 2.5 mM MgCl_2,_ 0.3 units of iTaq DNA polymerase (Biorad) and 0.2 µM forward–reverse primer (IDT, Coralville, IA). Real-time quantitative PCR amplification and detection was conducted using an iQ5 real-time quantitative PCR detection system (Biorad) and the following protocol: cycle conditions of 3 min at 95 °C, 40 cycles of 10 s at 95 °C and 30 s at 59 °C. Dissociation curves were run for all the primers used in this study to determine the presence of any nonspecific amplification. The relative gene expression was measured using the week 0 peel tissue as the calibrator. ‘No reverse transcriptase’ and ‘no template’ negative controls were included in PCR amplification. Each sample was represented by two independent total RNA isolations converted into two separate cDNAs. Each cDNA sample included two replicates for PCRs. Therefore, four separate PCR amplifications (four replicates) were performed on each sample. PCR amplification was carried out in triplicate in a 96-well plate. The target gene expression was normalized to that of the internal reference gene (*MdActin*) using the 2^−ΔΔCt^ method (the comparative Ct method).^32, 33^

## Results

### Fruit physiological changes and the incidence of CO_2_ injury incidence under three different storage regimens

Table 1 summarizes the patterns of physiological changes of ‘Golden Delicious’ apple fruit during the 12-week storage period. These physiological indicators showed considerable differences under three storage regimens. Significantly improved fruit firmness retention was observed under the CA and CAP storage regimens as compared to that under the RA storage condition. Fruit IEC was unique to each individual storage regimen. Under RA storage regimens, ethylene was readily detected after 2 weeks of storage and increased continuously until its concentration peaked at 8 weeks with an average value of 74.3 p.p.m. Significant suppression of ethylene production was observed in the CA storage regimen, particularly for those fruit at the 2- and 4-week storage durations, and IEC was higher by the end of the 12-week storage duration. Under the CAP storage regimen, ethylene was essentially eliminated by the combined or synergistic effect of CA and the action of 1-MCP during the full storage period. No significant difference in SPI was demonstrated among the storage regimens until the 12-week storage; thus, starch degradation essentially continued regardless of the storage regimen. The CO_2_ injury symptom was detected on 2.5% of CA-stored fruit after 2 weeks and increased linearly to 25% by the end of the 12-week storage period. Less than 5% of fruits showed CO_2_ injury symptoms over the full duration period after an increase from 2.5% at 2-week storage in CAP-stored fruit, with the exception of the 8-week sample, which recorded 10%. As expected, the CO_2_ injury symptom described above was not observed in fruits under RA storage. Overall, these storage regimens have resulted in distinguishable fruit physiological changes, particularly between RA and CA storage regimen, and the development of CO_2_ injury was not exacerbated by 1-MCP in the ‘Golden Delicious’ fruits used in this study.

### Reads processing and data analysis

A total of 334.6 million 100-bp single-ended reads, or an average of close to 30 million reads per sample for 13 apple fruit peel samples, including three storage treatments and four postharvest sampling points as well as an at-harvest (or week 0) fruit sample, were generated by Illumina HiSeq2000. The high-quality reads (332.6 million) were mapped to the ‘Golden Delicious’ apple genome reference, consisting of 122 107 contigs^34^, by using the CLCGW (CLCBio). Up to 82% of the sequencing reads were mapped to the reference genome. Among the mapped reads, 88% were ungapped reads and 75% were mapped to a specific genomic location. A total of 27 121 gene models were assembled. Kal’s test was performed to identify the differentially expressed genes (DEGs) within each storage regimen using the *P*-value with adjusted FDR (FDR ≤ 0.001) as the cut-off value. RNA-seq data were deposited in Gene Expression Omnibus with the accession number GSE62103 (http://www.ncbi.nlm.nih.gov/geo/query/acc.cgi?acc=GSE62103) at the NCBI website.

### Distribution of DEGs per storage regimen during the 12-week storage

**Figure 1:**
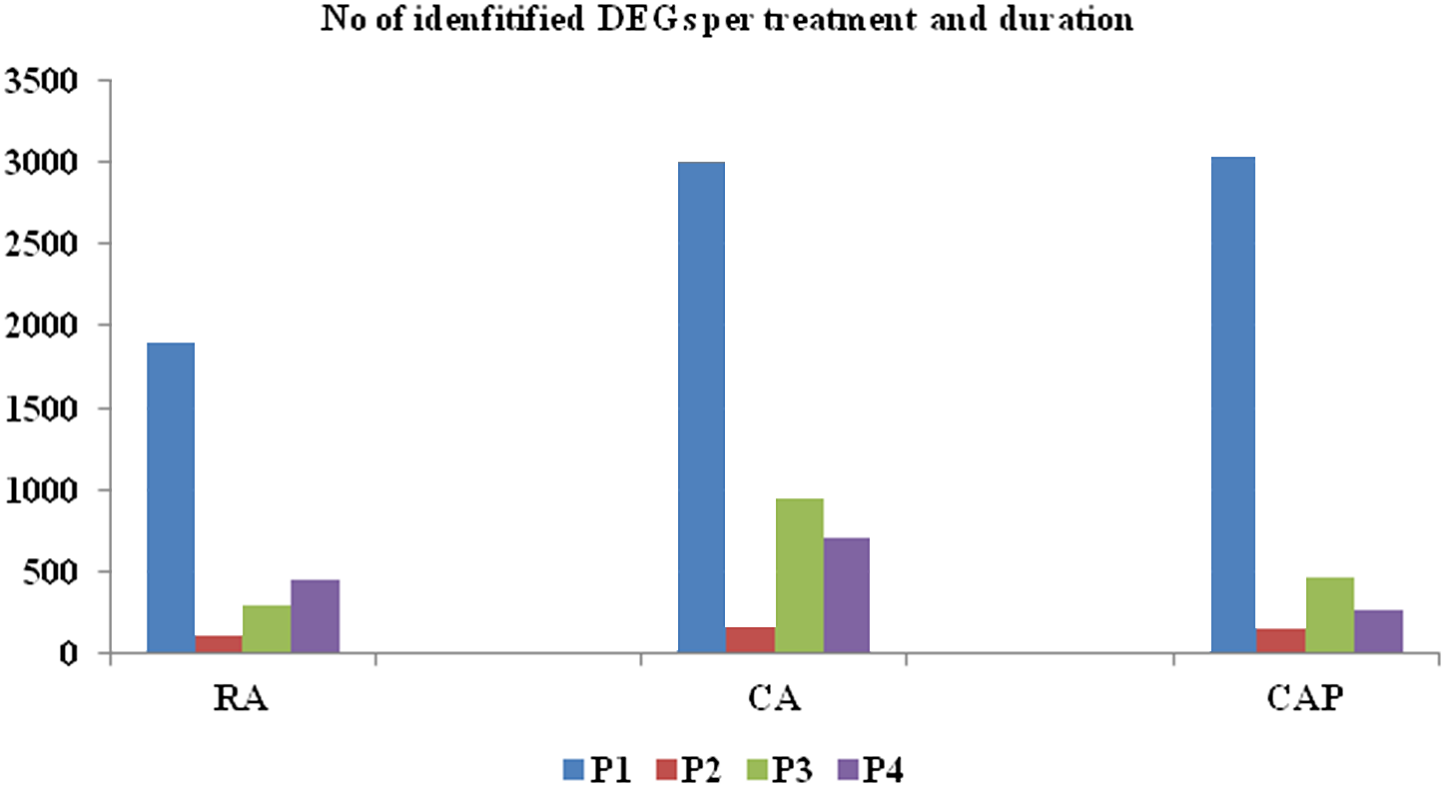
The distribution of identified DEGs per treatment and storage durations. Values on the y axis indicate the number of identified DEGs. See Materials and Methods for the details about the criteria of DEG identification.

**Figure 2:**
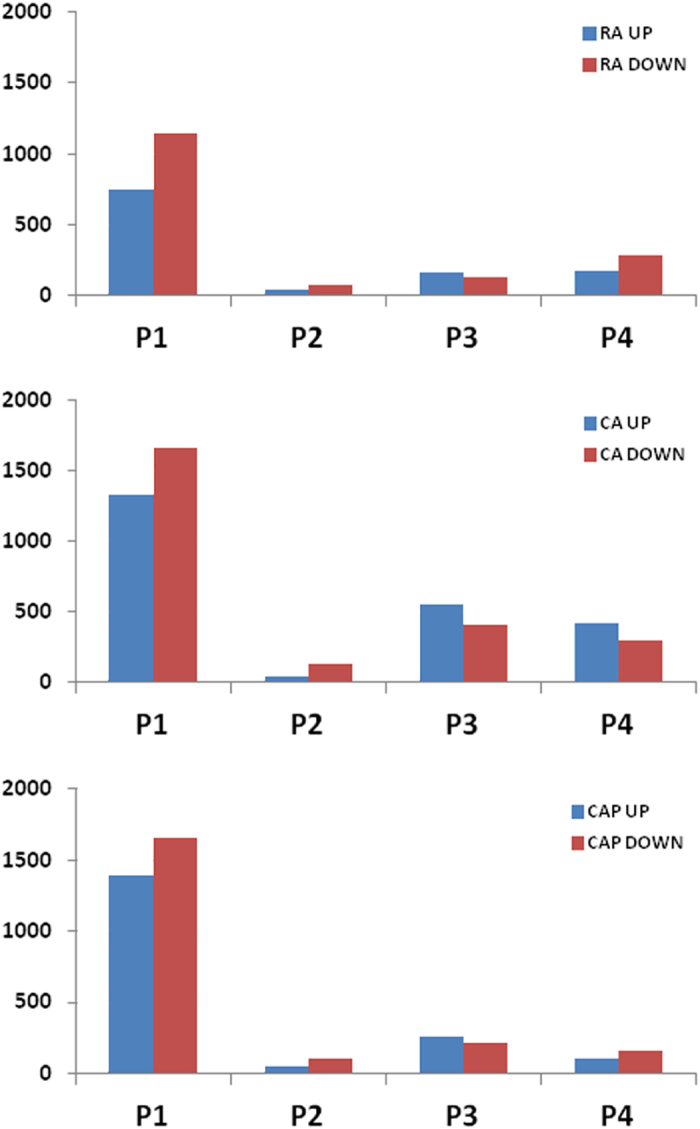
The regulation patterns of the identified DEGs per treatment and storage durations. Values on the y axis indicate the number of identified DEGs. See Materials and Methods for the criteria of DEG identification.

The overall distribution of identified DEGs between time points within the storage regimens indicated that the most radical transcriptomic change occurs during the first period of storage, or P1 (week 0 to week 2), regardless of the storage type. However, large number of DEGs were identified in CA and CAP compared to the RA storage regimen, suggesting that the additional transcriptomic change was attributed to the elevated CO_2_ concentration (Figure 1). By comparison, the numbers of DEGs in CAP were approximately half of those in CA, particularly after the 4-week storage, suggesting that the 1-MCP action alleviated the transcriptome changes induced by the CA storage condition. During the second period of storage, or P2 (week 2 to week 4), a significant decrease in the numbers of identified DEGs indicated a less agitated peel tissue transcriptome, possibly after acclimating to the cold storage temperature at 0.5 °C. In RA, the number of DEGs increased steadily from 2 weeks into the later storage periods (P3 = week 4 to week 8 and P4 = week 8 to week 12), although larger numbers of DEGs were identified for both CA and CAP storage regimens, and these numbers peaked at P3 for both the storage regimens. The overall shift in the direction of transcriptional regulation for these identified DEGs, i.e. either up- or downregulated expression patterns, appeared to be unique to each storage regimen (Figure 2). At P1 and P2 storage periods, more DEGs with downregulated patterns were observed for all three storage regimens. Then a higher number of DEGs with upregulated patterns were observed during P3 for each of the three storage regimens. However, this trend was more obvious for the CA storage regimen. On the other hand, in the RA and CAP storage regimens, the comparatively smaller numbers of DEGs and mostly downregulated expression patterns were common for all samples, except P3.

### Functional classification on the identified DEGs

Based on their annotation, DEGs were categorized into GO CC, MF, BP and KEGG Orthology (KO) pathway analysis. In total, 19, 114, 57 GO categories, respectively, and 20 KO categories were found enriched. More DEGs were mapped to MF than CC than BP. The number of enriched pathways was much greater for those DEGs identified in the CA storage regimen compared to those for RA and CAP storage regimens. The percentages of identified DEGs that were assigned to the GO and KO categories are listed in Table 2.

### Cellular components

**Figure 3:**
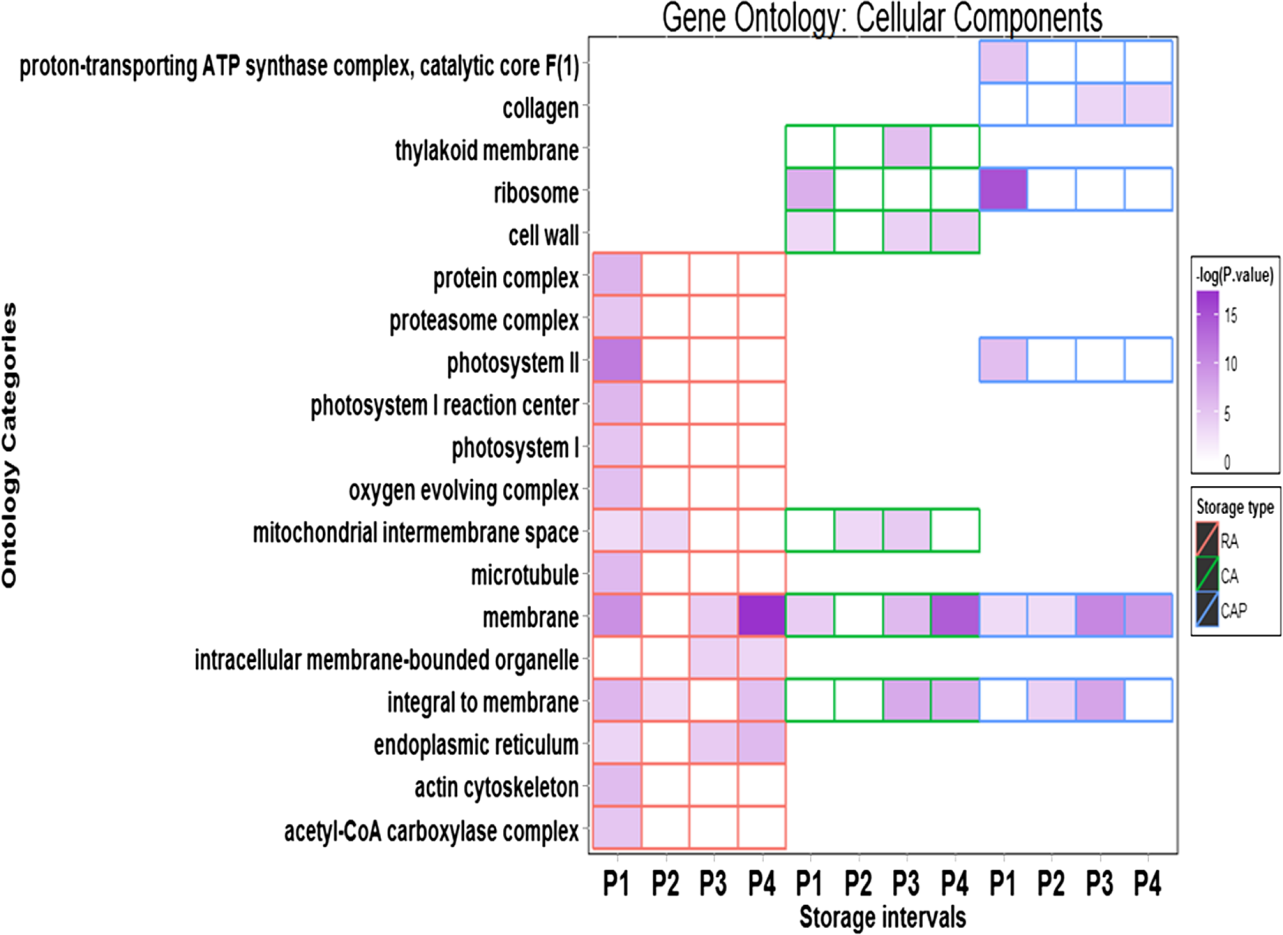
Differential enrichment among the three storage regimens for the selected subcategories within GO CC. Storage treatments are labelled with different colors: red for RA, green for CA and blue for CAP. The enrichment levels were expressed as –log (*P-*value) based on data GO anlysis using CLCBio software suit. For complete enrichment for subcategories, see the supplementary Figure S1.

At least nine relevant CC categories were shown to be differentially enriched among the storage regimens (Figure 3). ‘Membrane’ was systematically enriched in each storage regimen, particularly in P4. However, the enrichment levels descended more noticeably in the order of RA, CA than CAP. Therefore, it appeared that the elevated CO_2_ level and/or 1-MCP action impacted cellular function associated with the membrane. ‘Integral to membrane’ was also enriched in each storage regimen but with a lesser degree of enrichment compared to ‘membrane’. The enrichment of ‘protein complex’, ‘proteosome complex’ and ‘endoplasmic reticulum’ (ER) categories was only detected in the RA storage regimen, suggesting that these functions related to protein posttranscriptional processing were affected under the CA and CAP storage regimens. The ‘ribosome’ category was specifically enriched under the CA and CAP storage regimens, particularly at the P1 stage. The enrichment to ‘mitochondrial intermembrane space’ was detected in both the RA and CA storage regimens. Enrichment to ‘cell wall’ was only observed under the CA storage regimen.

### Molecular function

**Figure 4:**
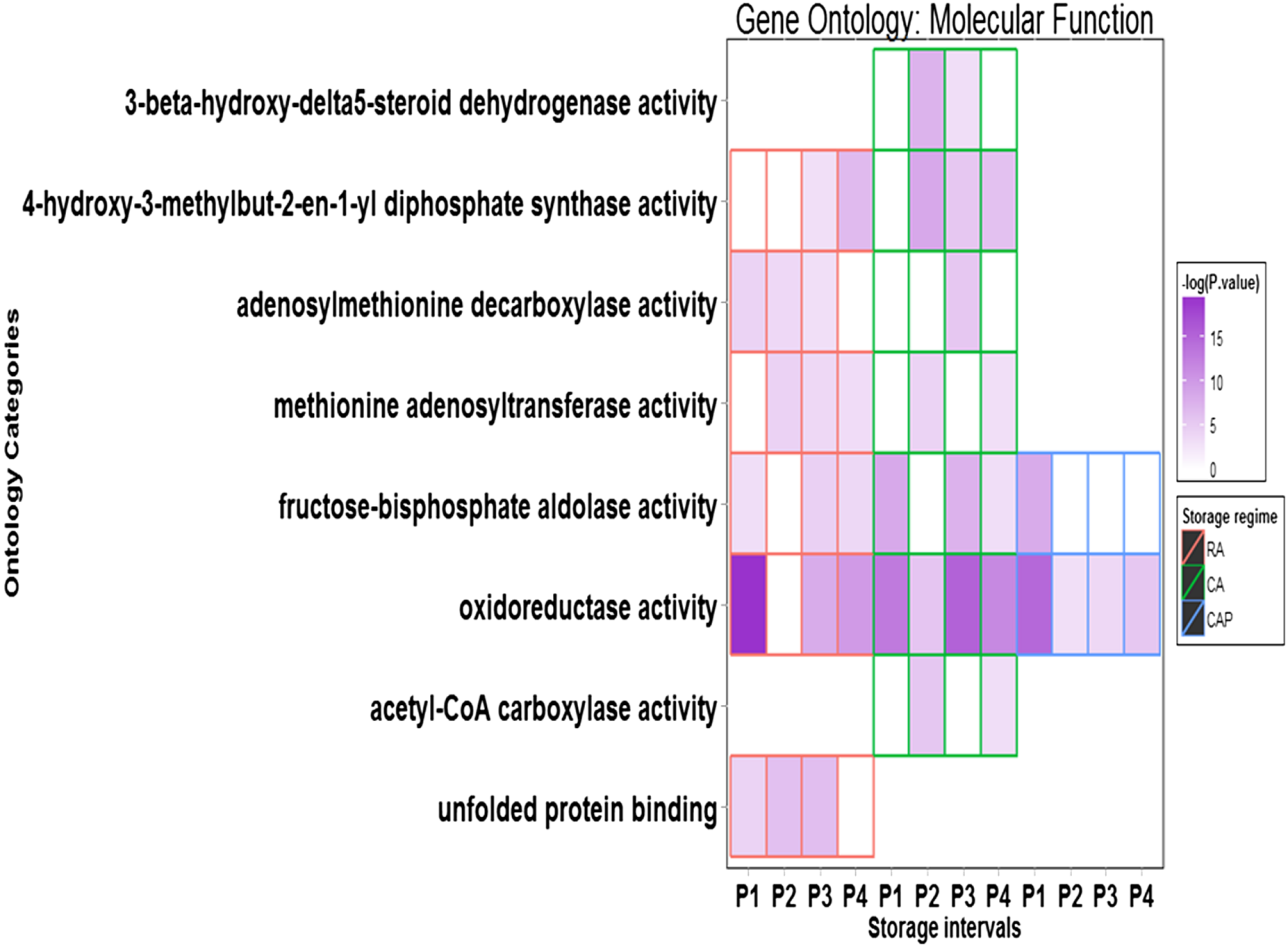
Differential enrichment among three storage regimens for selected subcategories within GO MF. Storage treatments are labelled with different colors: red for RA, green for CA and blue for CAP. The enrichment levels were expressed as −log (*P-*value) based on data GO anlysis using CLCBio software suit. For complete enrichment for subcategories, see the supplementary Figure S2.

For all three storage regimens, MF was the most enriched ontology, and considerable differences in the enrichment patterns for numerous categories were observed among the storage regimens (Figure 4; Supplementary Figure 1). The number of enriched MF categories for RA, CA and CAP storage regimens were 63, 70 and 59, respectively. It seemed that additional MFs were uniquely activated in the CA storage regimens. In addition, 1-MCP action in the CAP storage regimen alleviated the transcriptome agitation since there were fewer enriched MF categories compared to the CA regimen. The category of ‘oxidoreductase activity’, including those that oxidize specific chemical groups, was the most enriched category among the three storage regimens, and the CAP regimen was the least enriched. The ‘unfolded protein–binding’ category, which is related to protein processing, was found to be only enriched under RA condition. Substantial numbers of DEGs enriched with ‘transmembrane transporter activity’ and ‘transporter activity’ were specific to the CA and CAP storage regimens. The categories of ‘lipase activity’ functioning on lipid hydrolysis and ‘3-beta-hydroxy-delta5-steroid dehydrogenase activity’ for steroid biosynthesis were also found to be uniquely enriched in the CA storage regimen. The category ‘fructose-bisphosphate aldolase activity’ category, which represents a key mechanism in the glycolytic pathway, was enriched in all three storage regimens, though much higher enrichment levels were found in the CA regimen. The enrichments of ‘isoprenoid biosynthetic process’, ‘response to oxidative stress’, ‘response to stress’, ‘protein modification process’ and ‘carbohydrate metabolic process’ were found to be unique to the CAP storage regimen.

### Biological process

**Figure 5:**
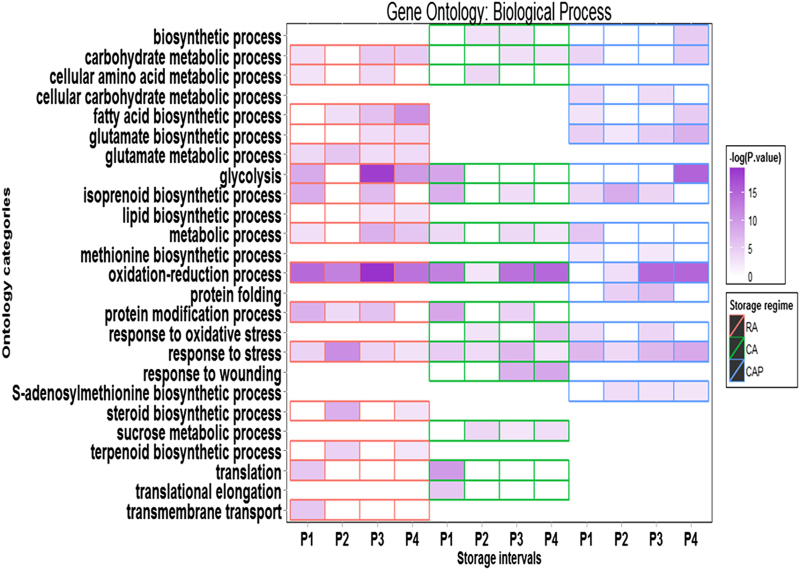
Differential enrichment among three storage regimens for selected subcategories within GO BP. Storage treatments are labelled with different colors: red for RA, green for CA and blue for CAP. The enrichment levels were expressed as −log (*P-*value) based on data GO analysis using CLCBio software suit. For complete enrichment for subcategories, see the supplementary Figure S3.

The most consistently enriched categories found within the BP ontology for all three storage regimens and at all time points were ‘oxidation–reduction process’, ‘glycolysis’, ‘isoprenoid biosynthetic process’ and ‘response to stress’ (Figure 5; Supplementary Figure 2). The enrichment for ‘response to oxidative stress’ category was shown in the RA and CAP storage regimens, but not in the CA storage regimen. The moderate enrichment of ‘glycolysis’ in the RA storage regimen was not detected prior to the P4 storage period. However, by contrast, this category was enriched in the CA regimen from P1 to P4 stages and peaked at the P3, while in the CAP regimen, a moderate gene enrichment level was observed and was detected only in the P1 stage. The ‘protein folding’ and ‘protein polymerization’ categories were uniquely enriched in the RA regimen, but ‘protein modification process’ was specifically enriched under both CA and CAP storage regimens. These observations together seemed to suggest that the processes related to proper posttranslational processing were essential during postharvest storage and were impacted by the CA and CAP storage regimens. Several BPs related to the biosynthesis of amino acid, lipid, terpenoid and steroid were specifically enriched in the CA storage regimen and so may be related to the response to the stress condition under this storage regimen. Also notably, both the ‘methionine biosynthetic process’ and ‘S-adenosylmethionine biosynthetic process’ categories, which are related to ethylene biosynthesis in plants, were enriched only in the RA storage regimen. This observation corroborated the suppression of whole-fruit ethylene production and values of fruit firmness in the CA and CAP storage regimens.

### KEGG pathway analysis

**Figure 6:**
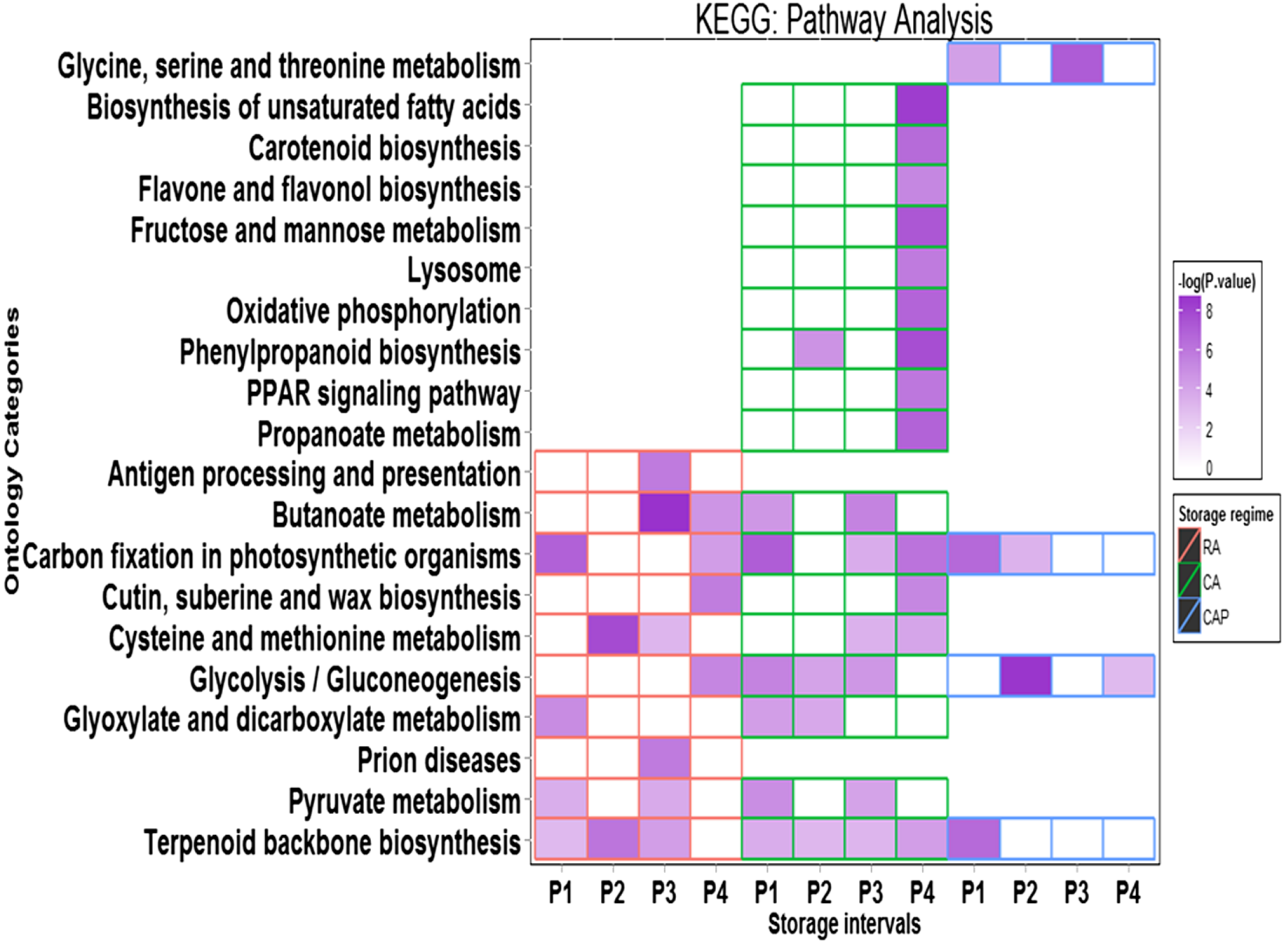
Differential enrichment for 20 pathways among the three storage regimen–based KEGG pathway analysis. Storage treatments are labelled with different colors: red for RA, green for CA and blue for CAP. The enrichment levels were expressed as −log (*P-*value) based on returned values from the KEGG pathway analysis at the KEGG Automated Annotation Server.

The differential enrichment patterns of individual KEGG pathways demonstrated significant shifts in the activities of biochemical pathways specifically in the CA storage regimen, compared to RA and CAP (Figure 6). DEGs identified from all storage regimens were mapped to the ‘glycolysis/gluconeogenesis’ pathway, but with different patterns. DEGs in RA mapped to this pathway were detected only in P4, while in CA, the enrichment to this pathway was from P1 till P3. Under the influence of 1-MCP, however, the enrichment of this pathway was delayed till the P2 stage, even though these fruits in CAP storage regimen were also under the same influence of elevated CO_2_ and reduced O_2_ level. As such, both GO BP and KO analyses identified in the glycolytic activities are the key impacted processes among the storage regimens. Enrichment patterns for ‘terpenoid backbone biosynthesis pathway’, which may be related to stress resistance mechanisms, were unique to each storage regimen; a stronger enrichment was detected in RA for most of the storage duration, except the last stage, a low but consistent enrichment level was found in CA during storage, while a strong enrichment was found in CAP but only in the P1 storage stage. ‘Cysteine and methionine metabolism’ was highly enriched in the RA regimen at the P2 and P3 storage stages but was minimally enriched under the CA regimen and was not enriched under the CAP regimen. Several pathways for the biosynthesis of secondary metabolites such as carotenoids and flavones were exclusively activated in the CA storage regimen; however, they appeared only in the last storage stage. The phenylpropanoid pathway was also unique to the CA storage regimen and was enriched in the P2 and P4 stages.

### Key cellular process revealed by transcriptome profiling analysis

#### Ethylene and jasmonate biosynthesis pathways

**Figure 7:**
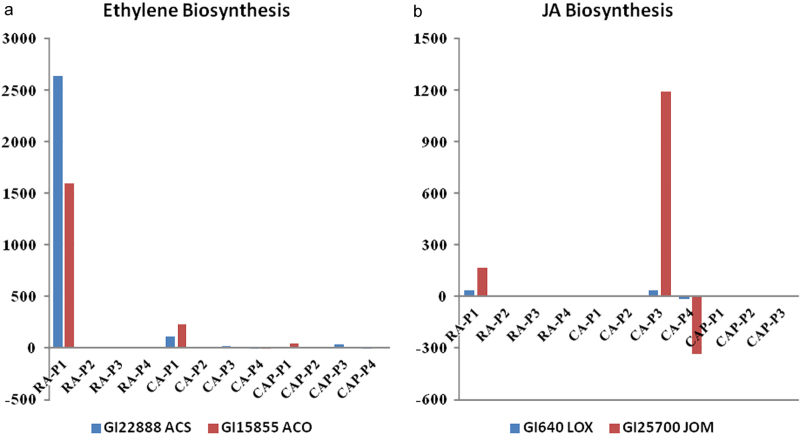
Differential regulation patterns of DEGs of ethylene and jasmonic acid (JA) biosynthesis pathways. Values on the y axis indicate the fold change of normalized transcript counts.

Within this RNA-seq data set, the distinguishable expression patterns of ethylene biosynthesis genes corresponded with each individual storage regimen. In the RA regimen, both *MdACS1* (GI 15 855) and *MdACO1* (GI 22 888) showed strong upregulation in P1, with 2638- and 1602-fold increase of transcript counts, respectively (Figure 7). In the CA storage regimen, the expression of *MdACS1* and *MdACO1* genes were still upregulated but with a suppressed degree compared to that in RA, i.e. with 106- and 226-fold increase of transcript counts for *MdACS1* and *MdACO1*, respectively. In the CAP storage regimen, the change of *MdACS1* transcripts was not detected, and only a 39-fold increase of *MdACO1* transcripts was detected, or approximately three times less compared to CA. In the later stages (P2 to P3), only a minimal upregulation or even down-regulation for both *MdACS1* and *MdACO1* was associated with these storage regimens. The regulatory patterns of these two key ethylene biosynthesis genes were consistent with the trends of measured IEC for each of the three storage regimens (Table 1). The direct effect by either CA or 1-MCP treatment on JA signaling pathway in apple fruit has not been reported previously. Based on this RNA-seq data set, a DEG annotated as jasmonate O-methyltransferase (GI 25 700) and linoleate 9S-lipoxygenase 1 (GI 640) was upregulated, with 36.8- and 2.3-fold increase of transcript levels, respectively, under RA condition at P1. Their induction of expression under the CA storage regimen was delayed until the P3 storage stage and with a stronger upregulation of 1192- and 37.6-fold increase at their transcript counts, respectively. The differential expressions for these two jasmonate biosynthesis genes were not detected under the CAP storage regimen.

#### Glycolysis

The differential enrichment to ‘glycolysis’ category was detected among the storage regimens based on GO BP analysis (Figure 5). A group of 16 DEGs enriched to this cellular process in RA, but was not enriched until the P4 stage (Table 3). Under the CA and CAP storage regimens, about double the numbers of DEGs enriched ‘glycolysis’ from the P1 stage, and particularly many of them had extended activity until the P4 stage but only in the CA regimen. Moreover, several of these DEGs were regulated with double-digit changes in transcript abundance, and multiple genes from the same gene family were regulated simultaneously under both CA and CAP regimens, but not under the RA regimen. These observations indicated that enhanced activities on ‘glycolysis’ were due to the increased CO_2_ and reduced O_2_ levels under CA storage. Furthermore, the data also showed that application of 1-MCP limited the enrichment only to the P1 stage. As shown in Table 4, a gene encoding ‘fructose-bisphosphate aldolase’ (GI 17 856) was identified with upregulated patterns under all three storage regimens; in RA, its fold change was approximately 6.3 and was delayed until the P4 stage, while a much higher transcript level was found under the CA and CAP storage regimens at the P1 stage, with 52.7- and 41.4-fold changes, respectively.

#### Protein folding and protein modification

The contrasting enrichment patterns between RA and CA storage regimens on the categories of ‘protein folding’ and ‘protein modification processes’ were detected based on the results from GO (BP) analyses. In the RA regimen, examination of the detailed regulation patterns of individual genes in ‘protein folding’ indicated that all of them were downregulated at the P2 and P3 stages (Table 4). On the other hand, double the numbers of genes were mapped to ‘protein modification process’ under the CA and CAP storage regimens during storage compared to those found in RA storage, though primarily limited to the P1 stage (Table 5). Putting these results together, it seems to suggest that normal protein folding process was impacted by CA storage regimen, and 1-MCP affected the differential expression of some of these genes associated with these categories. Thereby, removal of abnormally folded proteins, primarily by E3 ligases associated with the ubiquitin–26S proteome pathway, becomes a necessary process in order to maintain cellular integrity and control stress threshold levels.

#### Validation of the expression patterns of selected DEGs by quantitative reverse transcription–PCR

**Figure 8:**
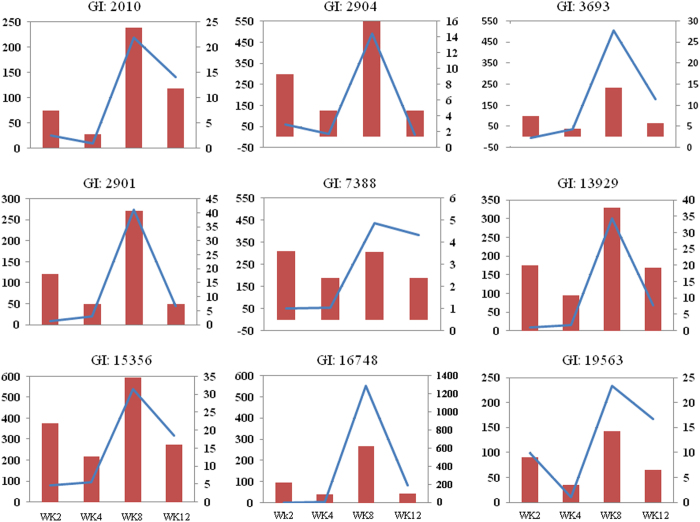
Validation of the expression patterns by quantitative reverse transcription–PCR for selected DEGs from RNA-seq analysis. The x axis represents the storage durations from left at 2, 4, 8 and 12 weeks. The values of y axis on the left side represent the fold changes, as indicated by the red bars, of gene expression level in relation to week 0 sample based on transcript counts from RNA-seq data set; while the values of y axis on the right side represent the relative expression level using quantitative reverse transcription–PCR, as indicated by the blue line, compared to the values of week 0.

The expression patterns for nine DEGs identified by RNA-seq–based transcriptome analysis from this study were validated by an independent approach of quantitative reverse transcription–PCR (Figure 8). Although different algorithms were used in quantifying their expression levels, a rather comparable dynamic of gene expression patterns was observed by both approaches for most of the selected genes, except the values at week 2 or week 4 for a few genes. Gene-specific primer sets, reference numbers in the apple genome database and the RNA-seq data are shown on Table S7.

## Discussion

The impacts of three different storage regimens on apple fruit physiology were demonstrated based on fruit firmness retention, fruit IEC levels and SPI. Suppression or elimination of climacteric ripening–related ethylene production was associated with the CA and CAP regimens, compared to the physiological indicators for fruit under the RA storage regimen. Better retention of fruit firmness under CA and CAP is almost certainly the result from the altered ethylene production. The external CO_2_ injury symptom on ‘Golden Delicious’ fruit under the CA regimen was shown on 2.5% of fruits at 2-week storage and increased to 25% at the end of 12-week storage. Notably, a reduced incidence level was observed in fruits under the CAP regimen compared to fruits under the CA regimen. This observation suggested that ‘Golden Delicious’ fruits are not sensitive to 1-MCP treatment and thus did not cause the aggravated CO_2_ injury symptom as found in the ‘Empire’ variant.^4, 17, 19^ Thus, it is evident that the aggravated effect of CO_2_ injury development by 1-MCP is an apple fruit cultivar-specific response. The abnormal patterns for 8-week and 12-week samples under CAP storage regimen on their CO_2_ rate were most likely caused by a mechanical failure in either of the individual chambers in holding the CO_2_ level, as fruit were randomly assigned for each treatment or time point.

The sudden transition of harvested fruit at ambient temperatures to storage temperatures at 0.5°C apparently resulted in a ‘cold shock’ in the apple fruit, as reflected by the strong transcriptome change in P1 for all storage types. Nevertheless, between the two different CA storage regimens, the larger numbers of DEGs compared to the RA regimen indicated additional stress factors related to elevated CO_2_ and reduced O_2_ levels. Then, the significant reduction on the numbers of identified DEGs for all storage regimens at P2 suggested that the fruit peel tissues have adapted, or acclimated, to the cold temperature. For example, more than 3000 DEGs identified at P1 in RA storage regimen were reduced to less than 200 at P2, indicating the transcriptome was considerably stabilized. As storage duration progressed, the number of DEGs increased gradually from P2 to P4 in RA, but a more abrupt increase in the number of DEGs was observed in both the CA and CAP storage regimens. Also, more DEGs had an upregulated pattern under CA storage regimen at P3 and P4. These observations suggested that under the CA and CAP regimens, additional molecular processes were activated in an attempt to alleviate the stress condition and maintain cellular integrity. Noticeably, application of 1-MCP under the CAP storage regimen decreased the level of transcriptome change compared to those under the CA condition, as indicated by the smaller number of identified DEGs.

Ethylene biosynthesis is one of the most important pathways in ripening apple fruit and its regulation patterns could influence apple fruit postharvest storability.^35–38^ Therefore, manipulation of this pathway is the focal point of apple storage technologies. In plants, ethylene biosynthesis is part of the ‘cysteine and methionine metabolism’ pathway, and the observed DEG enrichment pattern of this molecular pathway correlated with the measured whole-fruit IEC levels for each storage regimen. Although the observed ethylene biosynthesis was from apple fruit peel tissue, it was reported that the pattern of ethylene production follows similar patterns in both the peel and cortex tissues.^39^ As a high-throughput approach RNA-seq can offer the panoramic view of the global changes of gene expression in a plant tissue.^22^ Yet, with its high-resolution profiling and the ability to accurately determine the dynamic range of the expression level, the data set also allowed in-depth analysis on the regulatory patterns for the targeted pathways and their respective DEGs. Indeed, the transcript profiles on two ethylene biosynthesis genes, i.e. *MdACS1* and *MdACO1*, closely paralleled the trends of the measured IEC levels for each storage regimen and time point (Table 1, Figure 7a). From more than 27 000 assembled transcripts, and over 2000 identified DEGs in this RNA-seq data set, the detailed regulations of these two ethylene biosynthesis genes among these storage regimens highlight the power of RNA-seq platform. In other words, this technology can provide not only wide-angle transcriptome profiles but also the zoomed-in view of individual transcript levels and regulatory direction with great accuracy. The demonstration of delayed but enhanced activation of two JA biosynthesis genes is novel, as no available information is currently available regarding the influence of elevated CO_2_ and 1-MCP treatment on the jasmonate biosynthesis pathway in apple fruit. Although the molecular mechanism influencing the JA pathway under CA and CAP regimens remains unknown, the crosstalk between ethylene and JA signaling on the regulation of abiotic stress and apple fruit ripening and quality has been reported.^40–42^

Functional annotation for the identified DEGs and their enrichment to GO BP and KO categories revealed specific transcriptome changes under different storage regimens and their potential association with the development of CO_2_ injury symptom. Activation of glycolysis process seems to be inevitable in the living plant tissue such as apple peel, as reported in other climacteric fruits such as pear and avocado, particularly under CA storage regimens.^43, 44^ The earlier and stronger enrichment of identified DEGs mapped to ‘glycolysis–gluconeogenesis’ pathway under CA storage regimens implicated the potential connection of this process to the CO_2_ injury development. It is possible that the accumulation of some toxic intermediate metabolites, such as triphosphates or dihydroxyacetone phosphate, produced from the glycolytic process could be a factor linked to the development CO_2_ injury. In future studies, functional analysis of the candidate genes in the glycolysis pathway could provide more definitive answers for their roles in developing apple external CO_2_ injury. Although the mechanism by which 1-MCP alleviates CO_2_ injury in ‘Golden Delicious’ remains elusive, a less enriched glycolysis pathway, i.e. the fewer identified DEGs and shorter activation periods, was found under the CAP storage regimen (Table 3). It is remarkable that the majority of the DEGs identified from both CA-P1 and CAP-P1 exhibited virtually identical fold change and regulatory direction of some glycolytic genes (up- or downregulation) (Table 4). This observation seems to support the assumptions that glycolysis was one of the primary responses to elevated CO_2_ and reduced O_2_, rather than to cold shock. As demonstrated by GO analysis, the enrichment to glycolysis process due to 1-MCP was reduced after P1 even under the same level of elevated CO_2_ and reduced O_2_ concentration. On the other hand, continuing strong enrichments were found under the CA storage regimen.

Similar to the observed expressed patterns of genes in the ‘glycolysis’ pathway, the transcript level and the regulatory direction for these individual genes were highly comparable between CA-P1 and CAP-P1. Such observations seemed to suggest that these genes were specifically responsive to elevated CO_2_ and reduced O_2_ levels rather than 1-MCP alone. The absence of enrichment for both ‘cell wall’ and ‘mitochondrial intermembrane space’ under the CAP storage regimen perhaps implicated missing activities and the slowing down of the cell wall degradation process and the delayed respiratory climacteric burst associated with the apple fruit ripening.^45, 46^ The cellular activities associated with the ripening process in apple fruit tissues were significantly reduced due to the abolishment of ethylene production in the CAP storage regimens. As a result, reduced transcriptome changes such as a less enriched ‘glycolysis’ category and enhanced enrichment level in the ‘response to oxidative stress’ could alleviate the stress level in fruit peel tissue under the CAP regimen. Several molecular pathways related to the biosynthesis of phenylpropanoid, carotenoid and flavones were highly enriched under the CA condition at the P4 stage; however, at the later stage of storage and as CO_2_ symptoms developed on many fruits, it is more likely that the activation of these pathways was the result of the advanced cellular deterioration due to CO_2_ injury.

Removing abnormal proteins by selective protein breakdown through the ubiquitin–26S proteasome pathway is an essential housekeeping function in cells.^47^ It seems that under RA storage conditions cellular activities related to proper protein-folding process was less impacted, as indicated by the downregulated expression patterns of all DEGs mapped to ‘protein-folding’ process. For apple fruits stored in the CA or CAP storage regimen, on top of the stress related to cold shock, fruit cells have to cope with additional stress factors associated with elevated CO_2_ and reduced O_2_ concentrations. Under both CA and CAP storage regimens, the strong enrichment on the subcategory of ‘ribosome’ at the P1 stage may reflect the cellular effort to increase protein synthesis to mitigate the combined stress conditions of cold, elevated CO_2_ and reduced O_2_. At the same time in both CA and CAP storage regimens, no enrichment levels were detected for ‘protein complex,’ ‘proteasome complex’ and ER categories. They were, however, enriched in the RA storage regimen. It is possible that such additional stress factors have altered the cellular condition to a threshold level that perturbed protein-folding processes and could have led to the activation of an evolutionary-conserved physiological response known as ER stress.^48^ Taken together, while transcriptional regulations were made in response to all postharvest stress conditions, the necessary machineries and mechanisms for correctly processing these proteins were impacted by the elevated CO_2_ and reduced O_2_ and/or 1-MCP. These scenarios are supported by the differential enrichment patterns of genes related to ‘ribosome’, ‘protein folding’, ‘protein modification’ and other ER-related processes such as ‘protein polymerization’ and ‘unfolded protein binding’ among the storage regimens (Table 4). Moreover, cellular process to remove any unwarranted proteins is required to maintain cellular integrity and avoid the onset of organelle-level stress responses such as ER stress and the unfolded protein response pathways.^48, 49^

## Summary

Based on the identified DEGs, their functional assignment, the enrichment patterns to GO and KO categories, our transcriptome data set revealed tremendous adjustments of transcriptional activities per storage regimen and duration in the peel tissues of ‘Golden Delicious’ fruit. It appears that under the less stressful RA storage regimen, cells in fruit peel tissue can still function normally by preventing the irreversible disruption of cellular functions originated from cold shock and therefore maintain cellular integrity and relative normal physiology. However, the combined cold temperature and CA storage condition, either acting additively or synergistically as stress factors, caused additional transcriptomic changes in apple fruit peel tissue. The reduced effect on the overall transcriptome changes by 1-MCP application under the CAP storage regimen is intriguing. Perhaps the abolished ethylene responses and eliminated climacteric ripening process may slow down the overall physiological processes and therefore cause less interrupted cellular activities by stress factors originating from the CA condition. Under the CA storage regimen, multiple cellular processes were activated potentially to mitigate these stressful conditions. Comparative transcriptomics revealed that the imbalance of oxidative metabolism, enhanced glycolysis and suppressed ethylene biosynthesis were specifically associated with the CA storage regimen. On the other hand, the necessary BPs to reduce the damaging stress levels and maintain the cellular function such as posttranslational protein modifications and response to stress seemed to be compromised under the CA storage regimen. Overall, the data set from the current RNA-seq–based comparative transcriptomics revealed that different pathways and BPs were activated in the ‘Golden Delicious’ fruit peel tissues according to specific storage regimens. The identified DEGs and their specific enrichment patterns among the storage regimens allow outlining the possible molecular basis for the development of apple external CO_2_ injury symptom. The data set will be a valuable contribution for future hypothesis-driven studies aiming to identify specific genes and their roles in cultivar-specific responses to CO_2_ injury and/or 1-MCP treatment.

## Competing Interests

The authors declare no potential conflict of interest with respect to the authorship and/or publication of this paper. Mention of trade names or commercial products in this publication is solely for the purpose of providing specific information and does not imply recommendation or endorsement by the U.S. Department of Agriculture.

## Figures and Tables

**Table 1:** Physiological changes of apple fruit under three storage regimens and the incidences of CO_2_ injury.

Storage regimens	Storage duration (week)	IEC	Fruit firmness(lbs)	Fruit SPI(1–6 scale)	CO_2_ injury(%)
	0	0.1 ± 0.20	15.8 ± 1.25	3.5 ± 1.2	NA
RA	2	18.8 ± 5.4^d^	15.3 ± 1.1^a^	4.8 ± 0.5^a^	0
	4	38.1 ± 18.6^c^	15.5 ± 1.3^a^	5.0 ± 0.5^a^	0
	8	74.3 ± 17.2^a^	10.6 ± 1.4^b^	5.2 ± 0.3^a^	0
	12	50.6 ± 18.6^b^	10.0 ± 0.8^b^	5.9 ± 0.2^b^	0
CA	2	5.5 ± 14.2^e^	15.3 ± 0.8^a^	4.6 ± 0.3^a^	2.5
	4	0.2 ± 0.2^f^	15.8 ± 1.5^a^	5.0 ± 0.5^a^	5
	8	1.9 ± 3.3^f^	15.1 ± 1.6^a^	5.0 ± 0.4^a^	10
	12	7.0 ± 5.4^e^	15.4 ± 1.5^a^	5.4 ± 0.4^b^	25
CAP	2	0.1 ± 0.1^f^	15.3 ± 0.6^a^	4.6 ± 0.6^a^	2.5
	4	0.1 ± 0.1^f^	15.9 ± 0.9^a^	4.7 ± 0.8^a^	5
	8	0.2 ± 0.1^f^	15.3 ± 0.8^a^	5.1 ± 0.3^a^	10
	12	0.3 ± 0.5^f^	15.4 ± 0.8^a^	5.4 ± 0.5^b^	5

Fruit firmness, IEC and SPI were used to monitor the fruit physiological changes under different storage regimens. Values represent the average of 15 apples with standard deviation. CO_2_ injury rates were based on the assessment of 144 apples for each sample (time point/treatment). Means in columns with the same letter do not differ according *t*-test with significance level at *P* < 0.05.

**Table 2:** Percentage of the identified DEGs were assigned GO and KO terms.

Storage regimen	# of DEGs (FDR < 0.001)	CC (%)	MF (%)	BP (%)	PA (%)
RA	2738	11.3	51.5	20.7	15.1
CA	4479	8.7	59.4	27.3	37.7
CAP	4139	6.7	30.6	31.5	10.5

The number of DEGs (FDR < 0.001) for each storage treatment (Column 2) is depicted. Gene Ontology results for CC, MF and BP and KEGG Ontology Pathway (P).

**Table 3:** Identified DEGs mapped to ‘Glycolysis’.

Gene ID	Annotated function	RA/P4	CA-P1	CA-P3	CA-P4	CAP-P1
6155	Glucan endo-1,3-beta-glucosidase 7		2.9	−2.1		2.9
10108	Beta-enolase		−3.0			−2.6
9298	Enolase		3.1			5.1
24472	Enolase 1, chloroplastic	1.8	−3.2	3.2	−2.9	−3.3
17935	Pyruvate kinase isozyme G, chloroplastic		10.0	−2.8		10.8
10239	Pyruvate kinase	−1.5	2.0	−1.4	1.3	1.8
21474	Pyruvate kinase, cytosolic isozyme		2.3	−1.7		2.4
13054	Pyruvate dehydrogenase E1 component subunit	1.5	−2.4	3.2	−6.1	−3.7
18175	Pyrophosphate-fructose 6-phosphate 1-phosphotransferase		−2.4	2.6		−2.5
17258			−2.8	2.3		−3.2
4809	Phosphoglycerate kinase		−2.9			−3.9
3661	Phosphoglycerate kinase	−1.7	1.8		1.6	
7909	Hexokinase-4		4.8			4.7
16907	Nitrate transporter 1.3	1.3	−2.6	2.0	−3.2	−1.4
25484	Plastidial pyruvate kinase 2	1.4	1.8		−1.7	
14881	Hexokinase-2, chloroplastic	−3.1	2.1			
15054	Phosphoglycerate kinase, chloroplastic	−1.9	3.3	−2.0	1.3	3.1
17222	Probable fructose-bisphosphate aldolase, chloroplastic		−15.8			−21.0
5250		1.3	−1.7	1.6	−1.4	−2.2
1114			−1.6	1.8		−1.9
17856	Fructose-bisphosphate aldolase, cytoplasmic isozyme	6.3	52.7	−5.4	2.7	41.1
17855			1.5	−1.8		1.4
17783	Fructose-bisphosphate aldolase, chloroplastic		−22.6			−31.7
4665	Pyruvate kinase isozyme A, chloroplastic	1.5	−1.9	2.2	−3.0	−2.1
6800		1.7		3.0	−2.9	
2275		1.7		1.9	−2.8	−2.6
25307	Plastidial pyruvate kinase 1, chloroplastic	1.8		4.9	−4.4	
4780	Plastidial pyruvate kinase 2	1.6		2.6	−2.9	−2.2
18864	2,3-bisphosphoglycerate-dependent phosphoglyceratemutase			39.3	−42.3	
10409				3.5	−3.6	
796	Glucose-6-phosphate isomerase					−2.0
20509	Glucose-6-phosphate isomerase, cytosolic 2	−2.4	2.6	−2.1		2.6
9192	6-phosphofructokinase 5, chloroplastic					−5.3

Values are the Kal’s fold change–based transcriptome analysis using CLC Genomics Workbench with the criteria of FDR <0.001.

**Table 4:** DEGs mapped to the category of ‘protein folding’.

Gene ID	Annotated function	RA P2	RA P3
17298	Calreticulin		−2.1
6133	Calnexin homolog	−2.1	−2.1
15780	Endoplasmin homolog		−1.8
9363	Heat shock protein 90-2	−2.5	−1.7
1293	Peptidyl-prolyl cis-trans isomerase		−2.5
18166	Peptidyl-prolyl cis-trans isomerase	−2.1	
8241	Peptidyl-prolyl cis-trans isomerase		−1.5
17972	RuBisCO large subunit-binding protein subunit alpha (Fragment)		−1.7
575	RuBisCO large subunit-binding protein subunit beta, chloroplastic		−1.5
8893	Chaperone protein DnaJ		−2.4
144998	Chaperonin 60 subunit beta 3, chloroplastic	−1.5	
10034	20 kDa chaperonin, chloroplastic		−1.8
17063	DnaJ protein homolog ANJ1	−1.6	

Values are the Kal’s fold change–based transcriptome analysis using CLCGW with the criteria of FDR < 0.001.

**Table 5:** DEGs mapped to the BP category of ‘protein modification’.

Gene ID	Annotated function	RA P1	CA-P1	CA-P2	CA-P3	CAP-P1	CAP-P3
Gene 10351	E3 ubiquitin-protein ligase UPL4		3.0		−1.7	2.9	
Gene 1856	E3 ubiquitin-protein ligase UPL4		3.0			3.4	
Gene 10098	E3 ubiquitin-protein ligase UPL3		2.4		−1.6	2.5	
Gene 20635	E3 ubiquitin-protein ligase UPL3	1.6	3.1		−1.7	3.3	−1.4
Gene 26417	E3 ubiquitin-protein ligase UPL1	1.4	3.4	1.3	1.9	3.5	−1.5
Gene 21073	E3 ubiquitin-protein ligase UPL1	1.7	1.5			1.6	
Gene 3215	E3 ubiquitin-protein ligase UPL5	−2.1	−1.8			−1.3	
Gene 1404	E3 ubiquitin-protein ligase UPL6		1.6			1.7	
Gene 26418	E3 ubiquitin-protein ligase UPL2	1.4	3.2		−1.6	3.2	−1.4
Gene 21071	E3 ubiquitin-protein ligase UPL1	2.3	4.0	1.3	−1.3	3.7	−1.3
Gene 23215	E3 ubiquitin-protein ligase UPL5		3.2			3.2	
Gene 16038	Ubiquitin-activating enzyme E1 1	−3.7					
Gene 17428	Ubiquitin-activating enzyme E1 1					1.6	
Gene 24569	Protein-L-isoaspartate O-methyltransferase		2.6			3.4	
Gene 11080	Pentatricopeptide repeat-containing protein	−3.9	1.8	−1.4	−1.7	−1.9	−1.6
Gene 7798	NEDD8-activating enzyme E1 catalytic subunit		2.1		−2.0	1.9	

Values are the Kal’s fold change–based transcriptome analysis using CLC GW with the criteria of FDR <0.001.
