# Supplementary material for: Transcriptome changes in apple peel tissues during CO2 injury symptom development under controlled atmosphere storage regimens
Source: Hortic Res. 2015 Dec 23;2:15061–. doi: 10.1038/hortres.2015.61 (PMC4817516; doi:10.1038/hortres.2015.61)
Supplement: Supplementary Figures and Table [file hortres201561-s1.pdf]

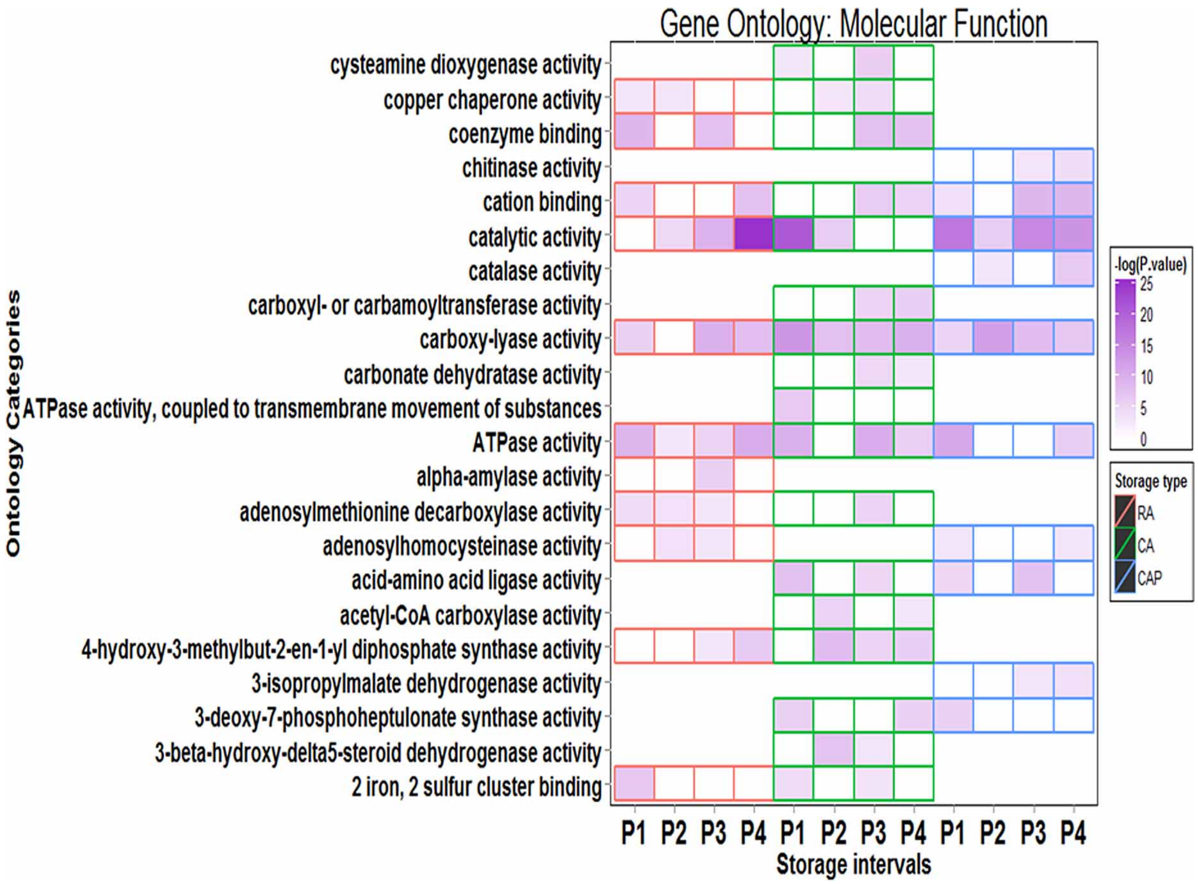

Supplementary Figure S1-1

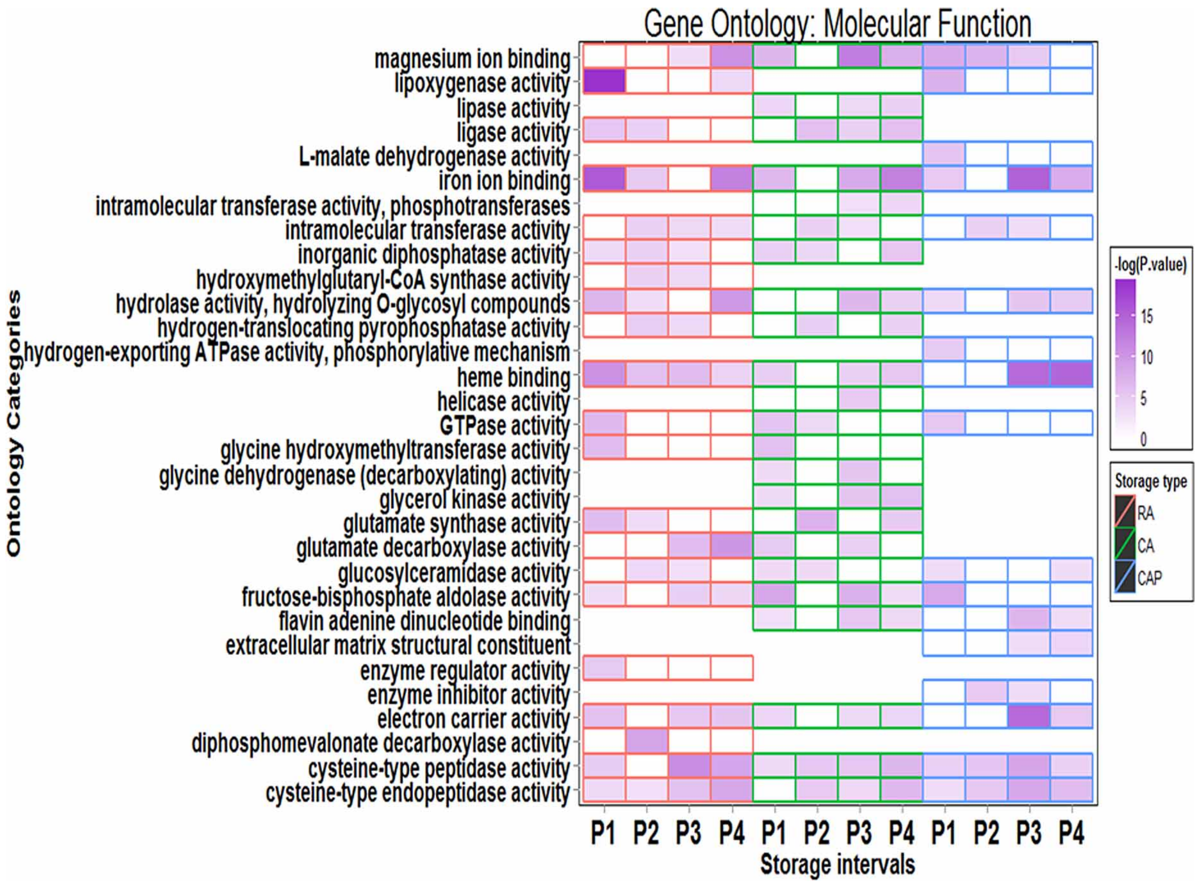

Supplementary Figure S1-2

© 2013 Nanjing Agricultural University

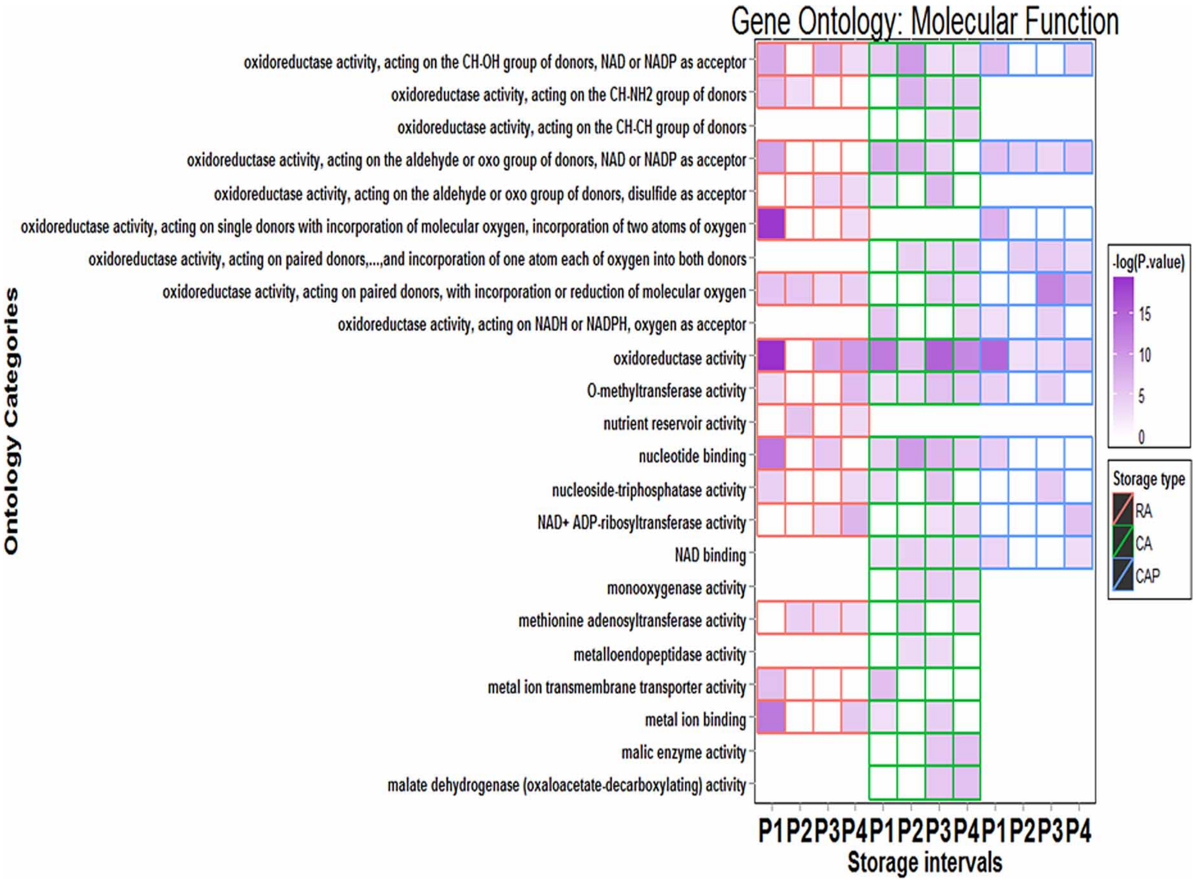

Supplementary Figure S1-3

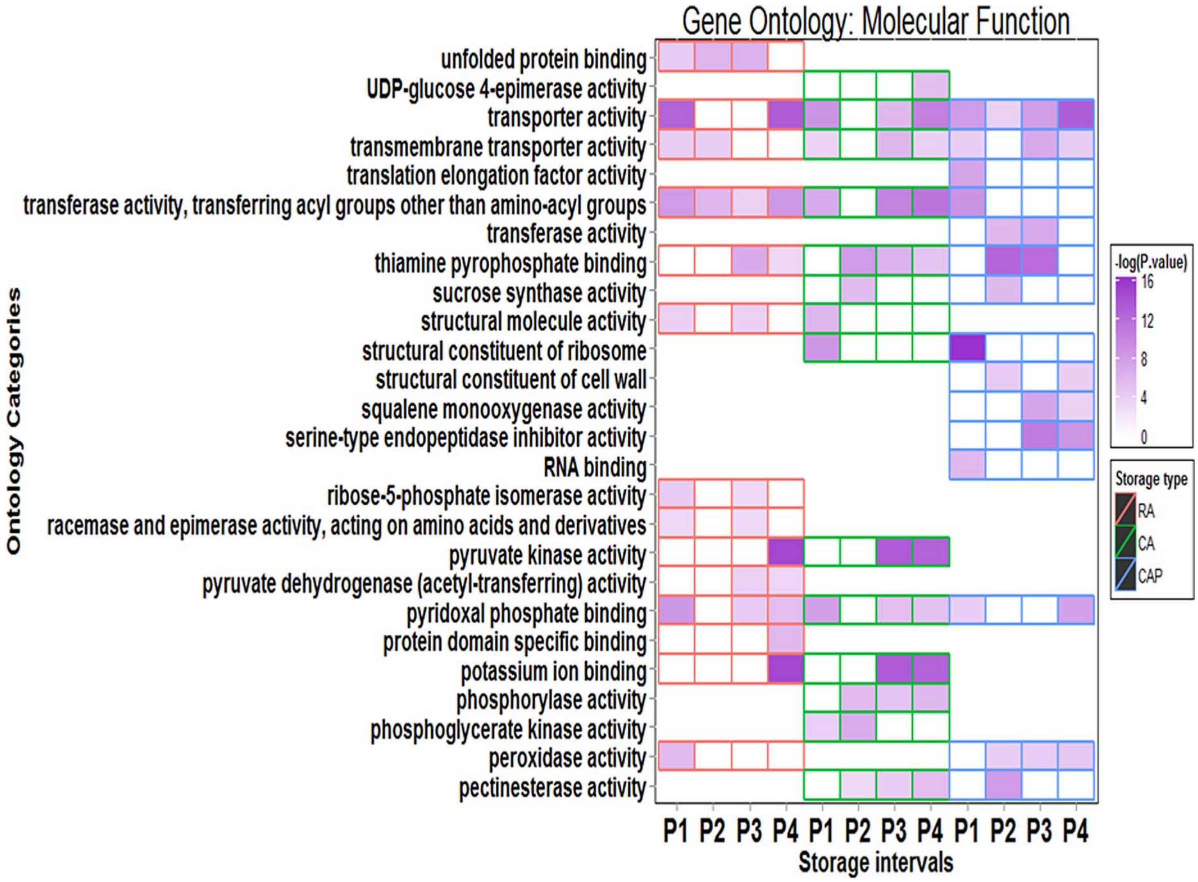

Supplementary Figure S1-4

© 2013 Nanjing Agricultural University

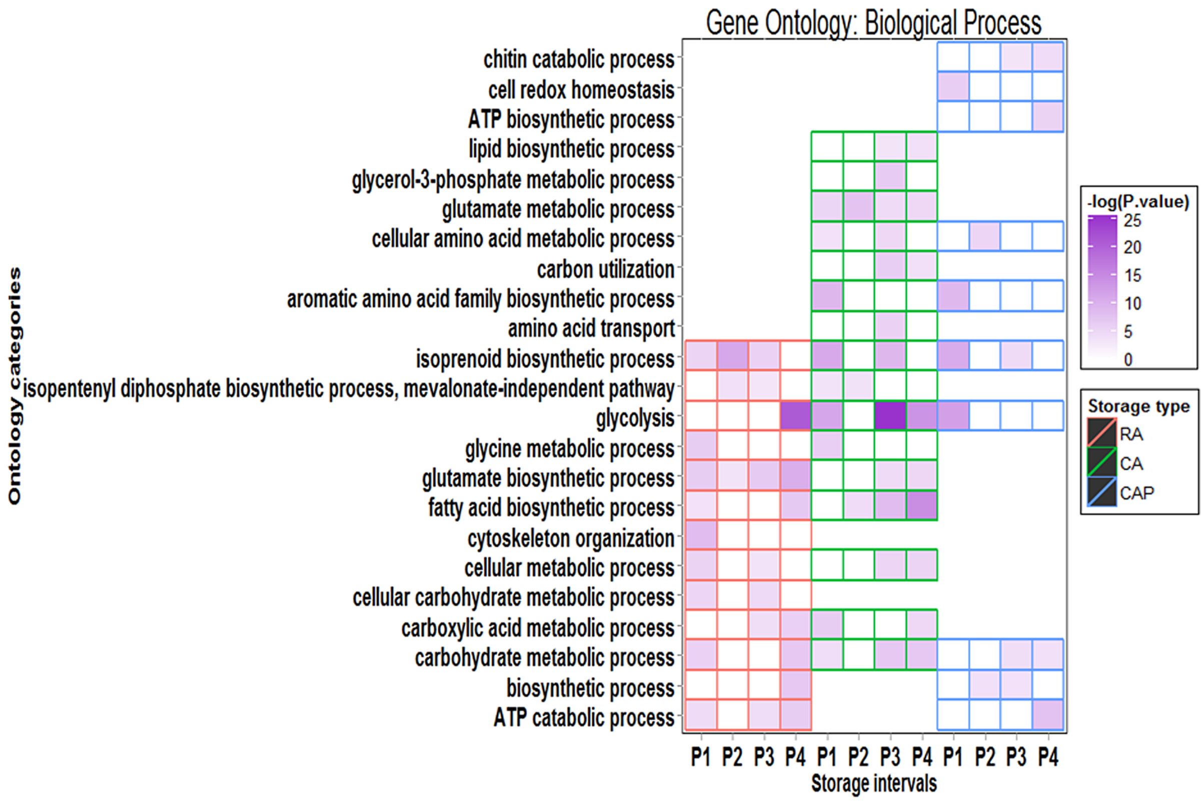

Supplementary Figure S2-1

© 2013 Nanjing Agricultural University

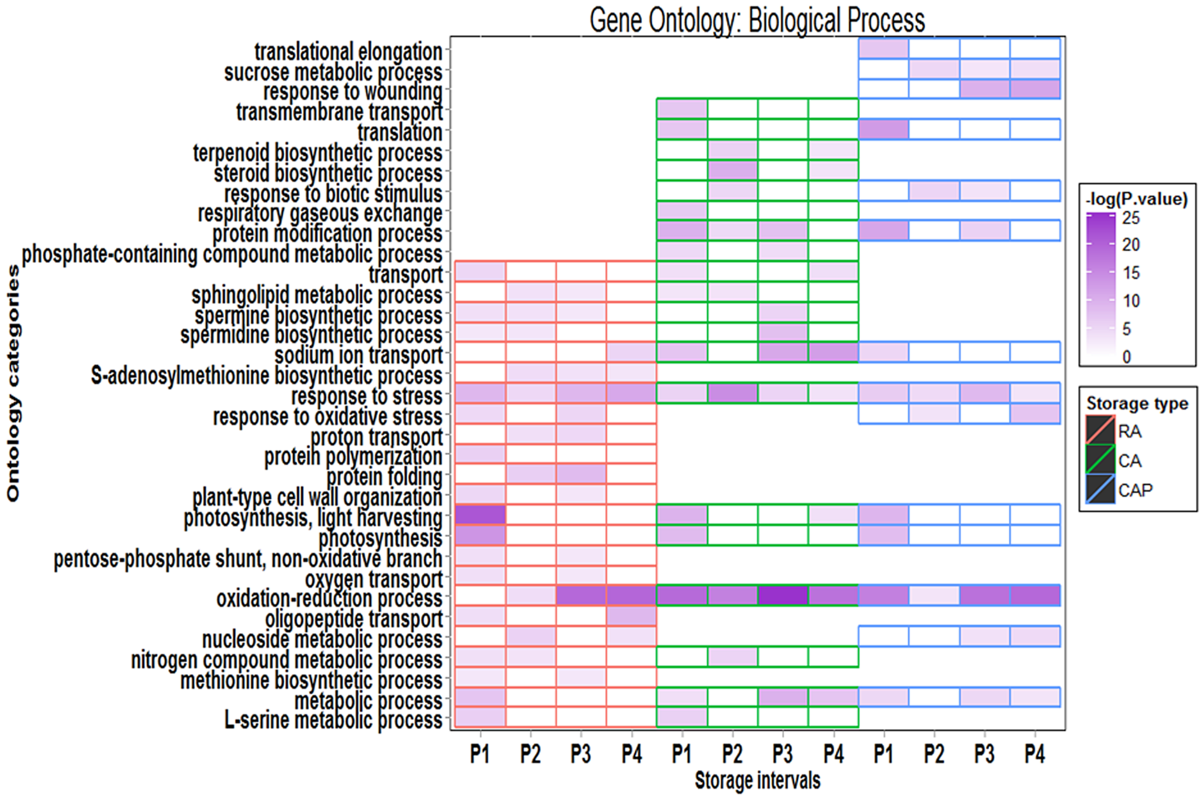

Supplementary Figure S2-2

© 2013 Nanjing Agricultural University

Table S1 Primer sequences used in data validation by qRT-PCR

Gene Identification	Gene model from apple genome	Primer Sequences
2010	MDP0000161311	Forward: TTAAGAGCTCAGAGGTGAC
		Reverse: CTCACCACCTTTCTTCTC
2901	MDP0000268045	Forward: GTCTACACAAAGGACTGG
		Reverse: TGAGGTGGAGTCTTGTAG
2904	MDP0000322755	Forward: GAACTGGTACTGCTATGG
		Reverse: GTCAGGTACTTGAGGATG
3693	MDP0000778598	Forward: AGTGCAGATGAACGATAC
		Reverse: GTACCACTAACAAGTCTTCC
7388	MDP0000136792	Forward: GCAGACTTCTATGTGGAG
		Reverse: CTGTGGTTCTACCATGTC
13929	MDP0000240828	Forward: GTACACACACAAGCCTTC
		Reverse: CTGACTTCTTGGTAGTGC
15356	MDP0000127019	Forward: AGATTTCTTAGCTCAGGTC
		Reverse: GCTAAGTGGCTAGAAGATG
16748	MDP0000466251	Forward: CAAGTGAGAAAGTTGCGTAAGA
		Reverse: AAAAGGTGGAGATGTGATGACT
19563	MDP0000129346	Forward: GGCTCGTACAAGTTTAGC
		Reverse: CACATGAGAAAGAGAGAGG

© 2013 Nanjing Agricultural University

Horticulture Research HORTRES201561_sup.3d 15/12/15 19:44:57
